# Genetic linkage maps for Asian and American lotus constructed using novel SSR markers derived from the genome of sequenced cultivar

**DOI:** 10.1186/1471-2164-13-653

**Published:** 2012-11-21

**Authors:** Mei Yang, Yanni Han, Robert VanBuren, Ray Ming, Liming Xu, Yuepeng Han, Yanling Liu

**Affiliations:** 1 Key Laboratory of Aquatic Plant and Watershed Ecology, Wuhan Botanical Garden, Chinese Academy of Sciences, Wuhan, Hubei, 430074, China; 2 Department of Plant Biology, University of Illinois at Urbana-Champaign, Urbana, IL, 61801, USA; 3 Key Laboratory of Plant Germplasm Enhancement and Specialty Agricultrue, Wuhan Botanical Garden, Chinese Academy of Sciences, Wuhan, Hubei, 430074, China

**Keywords:** Genetic linkage map, Genome sequence, *Nelumbo*, SRAP, SSR

## Abstract

**Background:**

The genus *Nelumbo* Adans. comprises two living species, *N. nucifera* Gaertan. (Asian lotus) and *N. lutea* Pers. (American lotus). A genetic linkage map is an essential resource for plant genetic studies and crop improvement but has not been generated for *Nelumbo*. We aimed to develop genomic simple sequence repeat (SSR) markers from the genome sequence and construct two genetic maps for *Nelumbo* to assist genome assembly and integration of a genetic map with the genome sequence.

**Results:**

A total of 86,089 SSR motifs were identified from the genome sequences. Di- and tri-nucleotide repeat motifs were the most abundant, and accounted for 60.73% and 31.66% of all SSRs, respectively. AG/GA repeats constituted 51.17% of dinucleotide repeat motifs, followed by AT/TA (44.29%). Of 500 SSR primers tested, 386 (77.20%) produced scorable alleles with an average of 2.59 per primer, and 185 (37.00%) showed polymorphism among two parental genotypes, *N. nucifera* ‘Chinese Antique’ and *N. lutea* ‘AL1’, and six progenies of their F_1_ population. The normally segregating markers, which comprised 268 newly developed SSRs, 37 previously published SSRs and 53 sequence-related amplified polymorphism markers, were used for genetic map construction. The map for Asian lotus was 365.67 cM with 47 markers distributed in seven linkage groups. The map for American lotus was 524.51 cM, and contained 177 markers distributed in 11 genetic linkage groups. The number of markers per linkage group ranged from three to 34 with an average genetic distance of 3.97 cM between adjacent markers. Moreover, 171 SSR markers contained in linkage groups were anchored to 97 genomic DNA sequence contigs of ‘Chinese Antique’. The 97 contigs were merged into 60 scaffolds.

**Conclusion:**

Genetic mapping of SSR markers derived from sequenced contigs in *Nelumbo* enabled the associated contigs to be anchored in the linkage map and facilitated assembly of the genome sequences of ‘Chinese Antique’. The present study reports the first construction of genetic linkage maps for *Nelumbo*, which can serve as reference linkage maps to accelerate characterization germplasm, genetic mapping for traits of economic interest, and molecular breeding with marker-assisted selection.

## Background

The genus *Nelumbo* Adans. comprises two living species, *N. nucifera* Gaertn. (distributed in Asia, Australia and Russia) and *N. lutea* Pers. (restricted to eastern and southern North America) [1,2]. The two species differ in features of their external morphologies, such as plant size, leaf shape, petal shape and color [1,2], and have significant genetic differences as revealed by DNA markers [3-7]. The species share the same chromosome number (2*n* = 16) and there is no interspecific reproductive barrier [8]. In Asia, lotus has been cultivated as a food crop for its rhizomes and seeds and as an ornamental plant for its magnificent flowers, in addition to its propagation for reasons related to its cultural and religious significance.

Although lotus is an important ornamental plant and a commercial crop, knowledge of the genomic constitution of *Nelumbo* species is limited. However, such information is a prerequisite for the identification of molecular markers linked to agronomic traits to facilitate breeding of improved lotus cultivars. A genetic linkage map is a powerful research tool for studies on plant genetics. Such a map may provide insights into genome organization, evolution and comparative genomics with related species [9]. From the perspective of improving crops, a genetic map is useful for map-based gene cloning, analysis of quantitative trait loci (QTL) underlying traits of economic importance, and molecular breeding using marker-assisted selection (MAS) [10,11]. Several DNA marker types, including random amplified polymorphic DNA (RAPD) [12-14], inter simple sequence repeats (ISSRs) [15,16], simple sequence repeats (SSRs) [3,6,7] amplified fragment length polymorphisms (AFLPs) [4,5], and sequence-related amplified polymorphisms (SRAPs) [17], have been developed for *Nelumbo*. The high degree of genetic diversity with *Nelumbo* revealed by these DNA markers facilitates the development of genetic linkage maps. Linkage maps have been constructed for many species, but as yet no genetic map has been developed for *Nelumbo*.

Simple sequence repeats are tandem repeats of one to six nucleotides present in all eukaryotic genomes [18]. Given their codominant inheritance, high polymorphism, and abundant distribution throughout genomes, SSRs have been used widely for genetic mapping, comparative analysis, and QTL analysis in plants [19,20]. Extensive efforts have been made to develop SSR markers in *Nelumbo* through genomic library screening using probes that contain repeated motifs and expressed sequence tags (ESTs) [3,5-7,21-23]. However, the total number of currently developed SSR markers (123 genomic SSRs and 39 EST-SSRs) is insufficient for genetic analysis in lotus. Moreover, such experimental approaches to developing SSR markers are laborious, time-consuming and costly. Therefore, there is a strong demand for additional SSR markers for lotus genome research and breeding. With the ever-increasing number of DNA sequences available in public databases, genomic sequences provide a more rapid and economic method for develop SSR markers. Based on SSRs developed from the genome sequence, high-density genetic linkage maps can be constructed [24-27].

Recently, we sequenced the genome of an ancient lotus cultivar, ‘Chinese Antique’, at 60 × coverage using a shotgun sequencing strategy, and the DNA sequences were assembled into 43,197 contigs. The total length of all contigs was 804 Mb, which represented 86.5% of the estimated 929 Mb lotus genome [28]. The availability of lotus genomic sequences provides an excellent opportunity to survey SSR motifs at a genome-wide level. The SSRs developed from the contig sequences can anchor corresponding contigs onto a genetic map and further establish direct links between genetic, physical, and sequence-based maps [25,29,30]. Therefore, a SSR-based reference genetic map is essential for assembly of the *Nelumbo* genome sequences and for integration of the genetic and physical maps of *Nelumbo*.

In the study reported herein, we identified SSRs from the contig sequences of the lotus cultivar ‘Chinese Antique’ and developed a new set of SSR markers to construct two genetic linkage maps. Our objectives were to: (1) assess the distribution of SSRs in the *Nelumbo* genome; (2) develop a large number of SSR markers and evaluate SSR polymorphism between *N. nucifera* ‘Chinese Antique’ and *N. lutea* ‘AL1’; (3) construct SSR-based reference linkage maps for Asian lotus and American lotus; and (4) anchor the SSR-associated contigs to the genetic map and facilitate assembly of the contig sequences.

## Results

### Identification and distribution of SSRs in the genome

A total of 86,089 SSR motifs with a minimum of five contiguous repeating units were identified within the contig sequences. Of the total SSRs identified, di- and tri-nucleotide repeat motifs were the most abundant repeat types, with frequencies of 60.73% and 31.66%, respectively (Table 1). Tetra- and penta-nucleotide repeats were the least frequent repeat types (5.77% and 1.21%). The number of each major SSR type identified within the lotus genome is summarized in Table 1. The most abundant dinucleotide motifs were AG/GA (31.08%) and AT/TA (26.90%). However, rare CG/GC motifs were identified. Among trinucleotide repeats, the AAG/AGA/GAA motif was the most common (11.79%), followed by the AAT/ATA/TAA (10.70%) and ATG/GAT/TGA (4.05%) motifs. GC-rich trinucleotide repeats were the least frequent. Of tetra-nucleotide repeats, AAAT/TAAA/ATAA/AATA was the most abundant, accounting for 26.47% of all tetra-nucleotide repeats, and followed by ATAC/TACA/ACAT/CATA (25.32%). Among pentanucleotide repeats, AGAAG/GAAGA/AAGAG/AGAGA/GAGAA motif was more common than other combinations. These data reflected AG/GA and AT/TA repeat motifs were the most abundant SSRs in the *N. nucifera* genome.

**Table 1:** **Distributions of the major SSR motifs identified from the genome of *Nelumbo nucifera *‘Chinese Antique’**

**Motif**	**Number**	**Frequency (%)**	**Range (bp)**	**Primers designed**	**Proportion (%)**
Dinucleotide	52282	60.73	16-110	44824	61.20
AG/GA	26753	31.08	16-98	23084	31.52
AT/TA	23158	26.90	16-110	19656	26.84
AC/CA	2367	2.75	16-72	2081	2.84
CG/GC	4	0.00	16-20	3	0.00
Trinucleotide	27258	31.66	15-141	22745	31.05
AAG/AGA/GAA	10146	11.79	15-141	8668	11.83
AAT/ATA/TAA	9212	10.70	15-102	7049	9.62
ATG/GAT/TGA	3485	4.05	15-84	3158	4.31
AAC/ACA/CAA	1160	1.35	15-90	962	1.31
AGG/GAG/GGA	1099	1.28	15-48	958	1.31
other	2156	2.50	15-138	1950	2.66
Tetranucleotide	4964	5.77	20-184	4276	5.84
AAAT/TAAA/ATAA/AATA	1314	1.53	20-164	1190	1.62
ATAC/TACA/ACAT/CATA	1257	1.46	20-184	1071	1.46
TATC/ATCT/TCTA/CTAT	858	1.00	20-84	741	1.01
TTTC/TTCT/TCTT/CTTT	740	0.86	20-60	596	0.81
TTAA/TAAT/AATT/ATTA	179	0.21	20-36	164	0.22
Other	616	0.72	20-144	514	0.70
Pentanucleotide	1045	1.21	25-145	964	1.32
AGAAG/GAAGA/AAGAG/AGAGA/GAGAA	253	0.29	25-80	211	0.29
AAAAG/AAAGA/AAGAA/AGAAA/GAAAA	109	0.13	25-45	106	0.14
TTTCC/TTCCT/TCCTT/CCTTT/CTTTC	104	0.12	25-145	102	0.14
AAAGG/AAGGA/AGGAA/GGAAA/GAAAG	98	0.11	25-70	90	0.12
ATATA/TATAA/ATAAT/TAATA/AATAT	95	0.11	25-95	90	0.12
Other	386	0.45	25-80	365	0.50

The SSR motifs represent variants of both strands of the DNA sequence (e.g., AAT/ATA/TAA includes the reverse complements ATT, TTA and TAT).

### Development of SSR markers and detection of polymorphism

All microsatellites were selected for SSR marker development. From the 86,089 SSR-containing sequences in contigs, 73,246 non-redundant SSR primer pairs were identified. Priority was given to di- and tri-nucleotide repeats, which accounted for 61.20% and 31.05% of the total number of SSR primers, respectively. Among the markers that contained dinucleotide repeats, the largest proportion was for AG/GA, followed by AT/TA motifs (Table 1). Only three markers that contained CG/GC motifs were identified because this motif was very rare in the genome sequences analyzed. Of the markers for trinucleotide repeats, the AAG/AGA/GAA motif was the most common (11.83%), followed by the AAT/ATA/TAA (9.62%) and ATG/GAT/TGA (4.31%) motifs.

Only 500 pairs of primers were tested for their amplification potential in the two parents and their six F_1_progenies. Primer sequence information, repeat motifs, amplicon sizes and polymorphism features for these 500 primers are listed in Additional file 1. Among these primers, 459 amplified at least one fragment in the genetic materials, and 73 primers that produced ambiguous fragments or smears were excluded from the genotype analysis of the F_1_ lines. Of the successfully amplified primers (386 pairs), the number of alleles per primer ranged from one to seven with a mean of 2.59, and a majority of them amplified two (218 pairs of primers) or three (82 pairs of primers) alleles. For 386 pairs of primers, 185 showed polymorphism among all eight lines, and 54 showed polymorphism in the two parents but no segregation in the six lines of the F_1_ population. Finally, 175 SSR primers were used to analyze the genotypes of all F_1_ progenies, which produced 450 markers with an average of 2.57 per primer (Table 2).

**Table 2:** Markers generated for the genetic mapping

**Marker**	**Number of primer used**	**Number of polymorphic primer**	**Maternal markers**	**Paternal markers**	**Total markers**	**Number of distorted marker**
Novel SSR	500	175	202	248	450	182
Previous SSR	95	33	27	41	68	31
SRAP	32	28	18	73	91	21
Total	627	238	247	362	609	234

### Analysis of genotypes in the F_1_ population

For 450 markers identified using the 175 novel SSR primers, 202 (44.89%) segregated in the female parent and 248 (55.11%) segregated in the male parent (Table 2). Thirty-three out of 95 previously published SSR primers were polymorphic among the parents and six F_1_ progenies (Additional file 2), which produced 68 segregating markers in all seedlings of the mapping population. The average number of markers per primer was 1.93. Among these 68 SSR markers, 27 (39.71%) segregated in the female parent and 41 (60.29%) segregated in the male parent (Table 2).

Of the 32 SRAP primers tested, 28 reproducibly amplified clear polymorphic fragments in the parents and all F_1_ progenies. A total of 91 SRAP markers were identified in the mapping population (Table 2). On average, there were 3.25 markers per primer combination, and the number of markers ranged from one to eight. Of the 91 SRAP markers, 18 (19.78%) originated from the female parent and the remaining of 73 SRAP markers (80.22%) originated from the male parent.

The goodness-of-fit of observed-to-expected allelic ratios was analyzed using the *χ*^2^ test. Of the total of 609 markers, 234 (38.42%) showed a significant deviation from Mendelian expectations (*P* < 0.05). 129 markers deviated toward the female parent and 105 markers toward the male parent. The 129 SSR distorted markers derived from the female parent showed no segregation in the F_1_ progenies, which indicated a high level of homozygosity in the female parent. The 234 distorted markers were excluded from the linkage analysis because they could lead to false linkage between some markers or linkage groups. The remaining 375 markers, which comprised 118 maternal markers and 257 paternal markers, showed the expected 1:1 segregation ratio and were used to establish the linkage groups.

### Construction of two linkage maps

A framework map was constructed using 305 SSR markers (Additional file 3). Only 32 markers were identified in the maternal map and were assigned to eight small linkage groups (LG1-F to LG8-F) with a total length of 362.52 cM. The number of markers per linkage group varied from three to seven. In contrast, 137 SSR markers were assigned to the paternal map, which consisted of eight major linkage groups (LG1-M to LG8-M) and three triplets (LG9-M to LG11-M) that covered a total length of 495.37 cM. The number of markers in LG1-M to LG8-M varied from eight to 27 with an average of 12.8 (Additional file 3).

**Figure 1:**
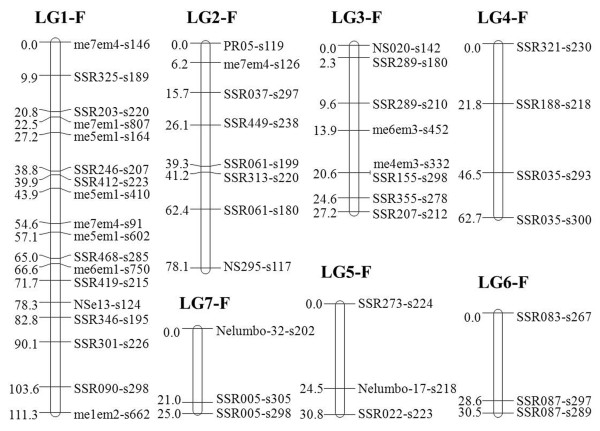
**Genetic linkage map of Asian lotus (*Nelumbo nucifera*) constructed with SSR and SRAP markers.** Map distances in centi-Morgans (cM) and marker names are shown on the left and right sides of each linkage group (LG1-F to LG7-F), respectively. The marker nomenclature corresponds to the primer name followed by “s” (abbreviation of “size”) and the size of product (in base pair).

**Figure 2:**
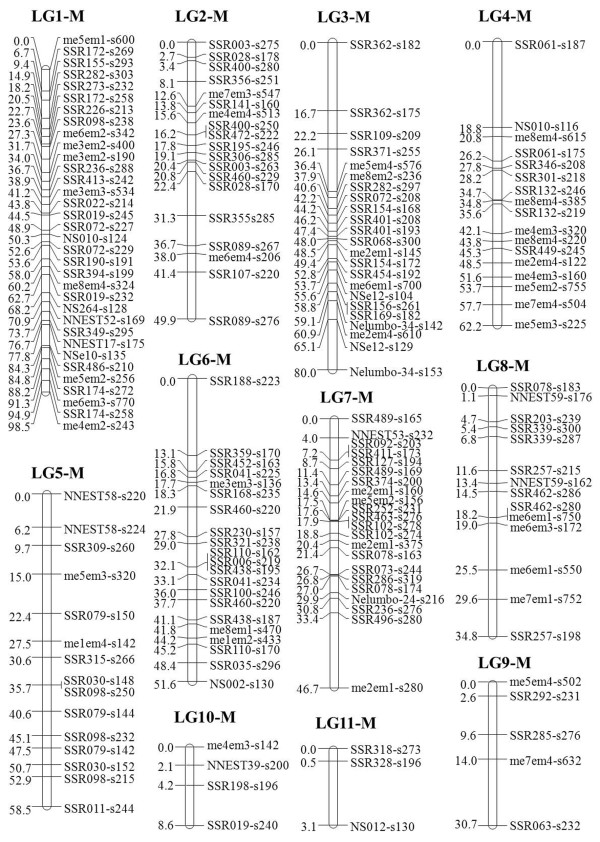
**Genetic linkage map of American lotus (*Nelumbo lutea*) constructed with SSR and SRAP markers.** Map distances in centi-Morgans (cM) and marker names are shown on the left and right sides of each linkage group (LG1-M to LG11-M), respectively. The marker nomenclature corresponds to the primer name followed by “s” (abbreviation of “size”) and the size of product (in base pair).

To increase map coverage, SRAP markers were added to the framework map to construct an integrated map. A total of 375 markers, including 268 novel SSR markers, 37 previously published SSR markers, and 70 SRAP markers were available for map construction. The addition of SRAP markers to the framework map allowed the SSR markers that were originally unlinked or floating to be placed as accessory markers. Using the SSR marker orders in the framework map as the preferred orders, 47 markers composed of 36 SSR and 11 SRAP markers were identified in the maternal map. These were assigned to seven linkage groups (LG1-F to LG7-F), with three to 18 markers in each group. The map spanned 365.67 cM with an average interval of 7.78 cM between adjacent markers (Table 3 and Figure 1). The paternal linkage map consisted of 177 markers distributed over 11 linkage groups (LG1-M to LG11-M). There were from three to 34 markers per linkage group. The paternal map spanned 524.51 cM with an average interval of 2.96 cM between adjacent markers. The genetic length of each linkage group varied from 3.14 cM (LG11-M) to 98.45 cM (LG1-M) (Table 3 and Figure 2).

**Table 3:** Number of markers and the length per linkage group of the integrated map, and the number of contigs corresponded to every linkage group

**Linkage group**	**Number of markers**				**Total length (cM)**	**Number of contigs**
	**Novel SSR**	**Previous SSR**	**SRAP**	**Total**		
Female (Asian lotus) map						
LG1-F	9	1	8	18	111.34	9
LG2-F	5	2	1	8	78.14	4
LG3-F	5	1	2	8	27.16	5
LG4-F	4	0	0	4	62.68	2
LG5-F	2	1	0	3	30.8	2
LG6-F	3	0	0	3	30.52	1
LG7-F	2	1	0	3	25.02	2
Male (American lotus) map						
LG1-M	20	5	9	34	98.46	19
LG2-M	16	0	3	19	49.89	12
LG3-M	14	4	5	23	80.02	12
LG4-M	7	1	9	17	62.15	4
LG5-M	11	2	2	15	58.49	6
LG6-M	17	1	3	21	51.58	11
LG7-M	16	2	4	22	46.67	13
LG8-M	8	2	4	14	34.76	5
LG9-M	3	0	2	5	30.72	2
LG10-M	2	1	1	4	8.62	3
LG11-M	2	1	0	3	3.14	3
Total	146	25	53	224	890.16	97

## Discussion

In the study, we analyzed the frequency of microsatellites identified from 43,197 contig sequences of *N. nucifera* ‘Chinese Antique’. The 804 Mb of genomic sequence analyzed covers 86.5% of the lotus genome. One SSR was detected in every 9.33 kb in the genome sequences. The frequency of SSRs in lotus is much lower than that reported in the genome sequences of *Brassica*[24,29,31,32] and rice [33], but higher than the estimated frequency of SSRs in the genome of sorghum [27]. In the contig sequences, dinucleotide repeat motifs (60.73%) were the most frequently detected, followed by tri- (31.66%), tetra- (5.77%) and penta-nucleotide (1.21%) motifs. The most abundant dinucleotide motif detected was the AG/GA type (31.08%), followed by AT/TA (26.90%) and AC/CA (2.75%). The most common trinucleotide motif detected was the AAG/AGA/GAA type, followed by AAT/ATA/TAA and ATG/GAT/TGA (Table 1). These motif types and their proportions in the lotus genome are in close agreement with the patterns observed in dicots such as *Arabidopsis*[34], *Brassica*[24,31,32] and papaya [35], and in the monocot sorghum [27], in which AT and AG combinations of base pair motif types are predominant. The distribution of SSRs in the lotus genome is different from those observed in humans and *Drosophila*, in which AC is the most frequent dinucleotide repeat motif, followed by AT and AG [18]. The GC repeat motif is extremely rare in eukaryotic genomes, except in rice [33,34], and was absent from the lotus genome. These data suggested that AG and AT motifs were rich in the lotus genome.

The pseudo-testcross strategy is suitable for construction of genetic maps using a F_1_ population and was applied first for genetic mapping in *Eucalyptus*[36]. Given that lotus is protogynous and cross-pollinated, a high level of heterozygosity is predicted for the genomes of *Nelumbo* species. With this prediction in mind, we created a F_1_ mapping population to construct genetic maps of *Nelumbo* in the present study. The two parents, *N. nucifera* ‘Chinese Antique’ and *N. lutea* ‘AL1’, diverge strongly in their geographical distributions and important morphological traits, such as plant size, and shape and color of the leaves and petals. The two parents also show considerable genetic differences [3-7], which was confirmed here by the high polymorphism (61.91%) detected using the novel SSR markers (Additional file 1). Of 609 markers identified in the mapping population, the markers originated from the female parent (40.56%) were less than those inherited from the male parent (59.44%).

234 markers (38.42%) showed distorted segregation at the *P* < 0.05 level (Table 2), which was higher than the distorted segregation ratio reported for *Dendrobium*[37], strawberry [38] and ryegrass [39]. Segregation distortion is a common phenomenon in mapping studies with a F_1_ population [37-39]. Distortion of segregation ratios may result from biological factors, such as genetic isolation mechanisms, chromosome loss, locus duplication, and gamete selection [40,41]. Nonbiological factors, such as scoring errors and sampling errors, also can lead to distortion in segregation ratios [42,43]. In the present study, both biological factors and technical problems may have caused the observed segregation distortion in the F_1_ population. The high level of homozygosity of the female parent ‘Chinese Antique’, which was unexpected, has contributed to the considerable segregation distortion in the F_1_ population. Hence, 129 of the markers with distorted segregation ratios derived from the female parent showed no segregation in the F_1_ progenies. Our recent analysis of the F_1_ population estimate heterozygosity to be 0.03% for ‘Chinese Antique’, and 0.37 for ‘AL1’ (unpublished data). Thus, the similarly low heterozygosity in the male parent aggravated the distorted segregation in the F_1_ population. We observed that 96 markers skewed to the male parent showed similar segregation ratios and were distributed in a group spanning 20.23 cM (data not shown).

Molecular marker selection for genetic mapping is crucial for the credibility of linkage maps. Due to their inherited characteristics, SSR markers have many advantages in genetic mapping. They can also serve as anchor markers during comparative mapping with related species [20]. However, the high level of homozygosity of the female parent precluded the availability of SSR markers assigned to the maternal map. Moreover, using a single type of DNA marker to construct a lotus linkage map would easily lead to uneven marker distribution and large intervals between adjacent markers. Therefore, in order to increase the coverage of the linkage map and reduce the gap between markers, SRAP markers were selected to analyze the genotypes of the F_1_ population. Given that SRAP primers usually amplify the genomic intron and exon regions of functional genes [44], they complement the use of SSR markers. When SRAP markers were added to the framework map constructed using SSR markers, the total length of linkage groups increased from 857.89 to 890.16 cM, and the average interval between two adjacent markers decreased from 5.08 to 3.97 cM. Moreover, SSR markers in LG4-F and LG6-F of the maternal framework map were incorporated into one linkage group LG1-F in the integrated map. No large gaps (> 25 cM) were detected in these genetic linkage maps (Additional file 3 and Additional file 4). Thus, to some extent, the application of SSR and SRAP markers increased the length of the linkage map and reduced the distance between two adjacent markers.

Using normally segregating markers, genetic linkage maps for *Nelumbo* were constructed successfully (Figures 1 and 2). The integrated linkage maps comprised 171 SSR and 53 SRAP markers. These SSR markers were derived from 93 novel SSR primers and 20 previously published SSR primers, and were anchored to 97 contigs of ‘Chinese Antique’ (Table 3 and Additional file 4). The contig-based SSR markers can anchor corresponding contigs onto a genetic map and further establish direct links between genetic, physical, and sequence-based maps [25,29,30]. This SSR-based linkage maps are important reference linkage maps with which to anchor contigs and assemble the genome sequence of *Nelumbo*. As a result, these 97 contigs were merged into 60 scaffolds (Additional file 4). In the following genome assembly work, on the basis of the order of SSR markers in linkage groups and single nucleotide polymorphism (SNP) markers identified by restriction-site associated DNA sequencing (RAD-seq) technology, 43,197 contigs of lotus were assembled into approximately 3,605 sequence scaffolds (unpublished data).

In theory, the number of linkage groups should be consistent with the number of haploid chromosomes. As a genus comprising only diploid species, members of *Nelumbo* have eight pairs of chromosomes. In the present study, 7 and 11 linkage groups were detected for the female and male plants, respectively, based on SSR and SRAP markers (Table 3). Thus, the numbers of linkage groups did not match with the haploid chromosome number. It may be inferred that some linkage groups in the paternal map should correspond to one chromosome, but were divided because a large interval existed between the linkage group. No markers were detected in the eighth linkage group used to create the maternal map. This may be attributable to the low degree of heterozygosity in the maternal genome. Many preliminary genetic maps for other plant species usually contain a higher number of linkage groups than expected [37,45]. Because the mapping population used in the present study was the F_1_ generation of the cross between ‘Chinese Antique’ and ‘AL1’, no recombination between homoeologous chromosomes was possible [46]. Therefore, the markers in the two parental linkage groups could not integrate into one group. The high quality of the DNA markers and the accuracy of genetic mapping were exemplified by the clear separation of linkage groups for ‘Chinese Antique’ and ‘AL1’ (Figures 1 and 2).

To improve the linkage map, the number of male and female linkage groups should be supposed equal to the haploid chromosome number, and the density of linkage group should be increased. The use of more advanced genotyping technology, such as high-throughput sequencing for SNP discovery at the whole-genome or -transcriptome scale, would aid the construction of a complete genetic linkage map. RAD-seq of multiple individuals using Illumina technology can identify and score thousands of SNP markers randomly distributed across the target genome [47,48]. The platform can effectively generate dense linkage maps for QTL analysis [49-51]. Our recent work involving the RAD-seq approach identified 6,622 SNPs in the present F_1_ mapping population and enabled construction of a high-density genetic map together with the SSR markers developed in the present study. This paternal map spanned 494.3 cM and comprised 4,031 markers (3,895 SNP and 136 SSR) in nine linkage groups (unpublished data). Using the common SSR markers as anchor markers, comparative analysis identified the collinearity of these linkage maps. The genetic maps generated in the present study can serve as reference linkage maps of *Nelumbo* species for efficient studies of comparative genetics, QTL analysis of traits of economic importance, and molecular breeding with MAS.

## Conclusions

We here present the first report of the construction of genetic linkage maps of *Nelumbo* with SSR markers derived from sequenced contigs. A total of 73,246 non-redundant SSR markers were identified at a genome-wide level. Of 500 SSR primers that were selected, 185 (37.00%) showed polymorphism among the two parental genotypes and six progenies of their F_1_ population. Using the normally segregating markers, two genetic maps for Asian and American lotus were constructed, which comprised 224 markers that spanned 890.16 cM. Ninety-seven contigs were anchored to linkage groups and were integrated into 60 scaffolds. The genetic maps will accelerate the exploitation of genetic resources, QTL analysis, map-based gene cloning, and molecular breeding with MAS in *Nelumbo*.

## Methods

### Plant materials

A segregating F_1_ population derived from a cross between *N. nucifera* ‘Chinese Antique’ (female) and *N. lutea* ‘AL1’ (male) was used to construct a linkage map. The segregating population consisted of 51 seedlings. Young leaves of these seedlings and their parents were collected for DNA extraction using the cetyltrimethylammonium bromide (CTAB) method described by Doyle and Doyle [52]. DNA samples were diluted to 50 ng μL^-1^, and stored at −20°C until use.

### Mining of microsatellites from the contig sequences and primer design

A total of 43,197 sequenced contigs of ‘Chinese Antique’ were screened for SSR motifs with the Msatfinder script implemented in PERL (http://www.genomics.ceh.ac.uk/msatfinder). All SSRs with repeat motifs of two or more base pairs and greater than five repeating units were recorded.

All microsatellites were analyzed for SSR marker development. To eliminate redundancy and to avoid designing redundant sets of primers for the same locus, the selected SSR-containing sequences were screened for redundancy by comparisons among the primer sequences and by means of BLASTN analysis against genomic sequences. The resultant contigs were parsed to design primer pairs, employing the standalone Primer3 (http://frodo.wi.mit.edu/) program with a MISA-generated Primer3 input file [53]. The primer length was set between 18 and 23 nucleotides with an optimum size of 20 nucleotides. The melting temperatures ranged from 50°C to 70°C with an optimum temperature of 55°C. The optimum GC content was set to 50% with a minimum of 30% and a maximum of 70%. The predicted PCR products ranged from 100 to 300 bp.

### Evaluation of SSR polymorphism

A total of 500 primers were selected from newly designed SSR markers and used to evaluate SSR polymorphism. Primers were designated ‘SSR’. Their sequences are listed in Additional file 1. All primers were synthesized by Invitrogen Biotech (Shanghai, China). All of the 500 SSRs, together with 95 previously published SSRs, were used to detect polymorphism among six F_1_ progenies and their parents. The previously published primers prefixed by ‘Nelumbo’, ‘NSh’, ‘PR’ and ‘NS’ were derived from genomic sequences of *N. nucifera*[6,21-23]. Primer pairs prefixed ‘NNEST’ and ‘NSe’ were developed from EST sequences by Pan et al. [7] and Kubo et al. [6]. The polymorphic SSR markers were applied for subsequent confirmation in all F_1_ lines.

All PCR amplifications were conducted in a 10-μL reaction mixture that contained 50 ng of DNA, 10 × PCR buffer, 2.0 mmol L^-1^ Mg^2+^, 0.2 mmol L^−1^ dNTPs, 0.8 mmol L^−1^ of each primer, and 0.5 U Taq DNA polymerase (MBI Fermentas Burlington, Ontario, Canada). A Bio-Rad MyCycler Thermal Cycler (Bio-Rad, California, USA) and the following program were used: initial denaturation at 94°C for 5 min; 35 cycles of 40 s at 94°C, 30 s at the appropriate annealing temperature, and 40 s of extension at 72°C; and a final elongation step at 72°C for 10 min.

All PCR products were separated in a 6% denaturing polyacrylamide gel that contained 7 mol L^−1^ urea and 0.5 × Tris-borate-EDTA (TBE) electrophoretic buffer. The gel was pre-run in 0.5 × TBE buffer at 75 W constant power for 30 min. After the samples had been loaded, the gel was run until the xylene cyanol tracking dye had passed through two-thirds of the gel. After electrophoresis, the gel was stained with silver nitrate solution. Allele sizes were compared with a 50-bp DNA Step Ladder (Promega, Madison, WI, USA). SSR markers were named according to SSR primer names followed by the sizes (in base pair) of the DNA fragments scored.

### Genotyping analysis by SRAP marker

Combination of the previously published ME forward primers (ME1 to ME8) and EM reverse primers (EM1 to EM4) for sequence-related amplified polymorphism (SRAP) marker were used to analyze the genotypes of the F_1_ population [44]. Each 10-μL PCR mixture consisted of 50 ng DNA, 10 × PCR buffer, 2.0 mmol L^−1^ Mg^2+^, 0.2 mmol L^−1^ dNTPs, 0.8 mmol L^−1^ of each primer, and 0.75 U Taq DNA polymerase. The PCR amplification procedure was as follows: 5 min of denaturation at 94°C; five cycles of 1 min of denaturation at 94°C, 1 min of annealing at 35°C, and 1 min of elongation at 72°C, then 30 cycles with the annealing temperature at 50°C; and a final elongation step of 7 min at 72°C. The amplified SRAP fragments were separated and visualized using the same procedure for the SSR fragments.

### Segregation analysis and construction of genetic linkage map

The genotypic data for the F_1_ population analyzed with SSR and SRAP markers were used for the construction of a genetic map of lotus. The linkage analysis was performed using JoinMap version 4.0 [54]. Two heterozygous alleles from either parent were expected to segregate at a 1:1 ratio. The observed and expected allelic ratios for all markers were compared using the chi-squared (*χ*^2^) test. Markers were excluded from the linkage analysis when they showed significantly distorted segregation (*P* < 0.05). Only the markers with a *P* value higher than 0.05 were used for further linkage analysis.

To construct a suitably robust map, we followed the strategy described by Yang et al. [55]. This involved first using SSR markers to construct a preliminary framework map, and then joining SRAP markers to construct an integrated map using the order of the SSR markers in the framework as preferred orders (the ‘fixed order’ function). During construction of the framework and integrated maps, maternal and paternal data sets were created using the function ‘Create Maternal and Paternal Population Node’ in the JoinMap program. Maternal and paternal data were used to construct maternal and paternal maps, respectively. The regression mapping algorithm was used for map construction. The threshold for goodness of fit was set to ≤ 5.0 with logarithm of the odds ratio (LOD) scores > 1.0 and a combination frequency < 0.35. Map distances were calculated using Kosambi’s mapping function, and denoted in centi-Morgans (cM).

## Competing interests

The authors declare that there are no competing interests.

## Authors’ contributions

MY carried out genetic mapping, analyzed the data, and drafted the manuscript. YNH participated in the marker development and polymorphism detected. RVB participated in the sequence analyses and the marker development. RM participated in the design of the study and helped draft the manuscript. LMX cultivated and provided the plant materials. YPH participated in genetic mapping and drafted the manuscript. YLL conceived the study, participated in its design and coordination, and helped to draft the manuscript. All authors read and approved the final manuscript.

## Supplementary Material

Additional file 1 **Primer sequence information, repeat motifs, amplicon sizes, and polymorphism features for novel simple sequence repeat (SSR) markers from the genome sequences of *Nelumbo nucifera *'Chinese Antique'.** Click here for file

Additional file 2 **Details on previously published simple sequence repeat (SSR) markers of *Nelumbo nucifera.*** Click here for file

Additional file 3 **Framework map constructed using simple sequence repeat (SSR) markers for *Nelumbo.*** Click here for file

Additional file 4 **Integrated map constructed using simple sequence repeat (SSR) and sequence-related amplified polymorphism (SRAP) markers for *Nelumbo.*** Click here for file
